# A network meta-analysis on the beneficial effect of medical expulsive therapy after extracorporeal shock wave lithotripsy

**DOI:** 10.1038/s41598-017-14862-3

**Published:** 2017-10-31

**Authors:** Tong-Xin Yang, Bang-Hua Liao, Yun-Tian Chen, Hong Li, Qing He, Qin-Yu Liu, Kun-Jie Wang

**Affiliations:** 1 Department of Urology, Institute of Urology (Laboratory of Reconstructive Urology), West China Hospital, Sichuan University, Chengdu, Sichuan P. R. China; 2 Department of Urology, The Second Affiliated Hospital of Kunming Medical University, Kunming, Yunnan P. R. China

## Abstract

We applied a newly introduced method, network meta-analysis, to re-evaluate the expulsion effect of drugs including tamsulosin, doxazosin, nifedipine, terazosin and rowatinex after extracorporeal shock wave lithotripsy (ESWL) as described in the literature. A systematic search was performed in Medline, Embase and Cochrane Library for articles published before March 2016. Twenty-six studies with 2775 patients were included. The primary outcome was the number of patients with successful stone expulsion. The data were subdivided into three groups according to duration of follow-up. A standard network model was established in each subgroup. In 15-day follow-up results, SUCRA outcome showed the ranking of effects was: doxazosin > tamsulosin > rowatinex > nifedipine > terazosin (88.6, 77.4, 58.6, 32.2 and 30.4, respectively). In 45-day follow-up results, SUCRA ranking was: tamsulosin > nifedipine > rowatinex (69.4, 67.2 and 62.6, respectively). In 90-day follow-up results, SUCRA ranking was: doxazosin > rowatinex > tamsulosin (84.1, 68.1 and 49.1, respectively). In conclusion, doxazosin and tamsulosin have potential to be the first choice for pharmacological therapy to promote the expulsion of urinary stone fragments after ESWL, with this doxazosin can improve the SFR in the long term, while tamsulosin may result more in accelerating the process of expulsion.

## Introduction

Extracorporeal shock wave lithotripsy (ESWL) represents an effective minimally invasive treatment for renal and ureteral stones greater than 5 mm in size^1^. However, the elimination of calculus disintegrated by ESWL depends on many factors, including stone fragment volume, location and renal collecting system anatomy, as well as ureteral status, such as oedema and spasm^2^. To improve the results of ESWL, many institutions have combined medical expulsive therapy (MET) for urinary stone treatment to obtain a better stone-free rate (SFR).

Medications for urinary calculus such as α-adrenoceptor antagonist or calcium channel blocker have been investigated as spasmolytic agents that would promote the expulsion of stones. Among these, tamsulosin and nifedipine have received the most attention. Both tamsulosin and nifedipine are believed to act on the ureteral muscle, causing relaxation and dilation of the ureter and facilitating the elimination of fragments^3–5^, while nifedipine also relaxes the ureteropelvic junction, improving the urine flow to the ureter^6^. However, among the clinical data related to MET, contradictory results have been reported. Recently, Pickard and colleagues^7^ reported a high-quality randomized controlled trial, concluding that tamsulosin and nifedipine were not effective at decreasing the need for further treatment for patients compared with expectantly managed ureteric calculus. Thus, whether MET can improve SFR after ESWL also requires re-evaluation.

Several randomized controlled trials (RCTs) studying the SFRs in different types of pharmacological therapy after ESWL have been reported^8–33^. However, most of these studies were designed to compare the clearance rates between one medication and control^8–10,12,13,15–20,22–32^. Moreover, in most studies, an arbitrary endpoint time was set to assess the stone clearance rate while ignoring possible variations of therapeutic effect before and after that endpoint time^8–14,18,19,21,23,25,26,28,29,31–33^. Accordingly, one RCT can precisely resolve the issue of whether a medication can improve the SFR compared with a placebo or watchful waiting after a certain period, but cannot provide a wide view of its expulsion effect throughout the therapeutic process, and cannot reveal the difference between medications either.

In this context, network meta-analysis is a useful method in which multiple treatments can be directly or indirectly compared even if they are not designed in the same RCT, but have the same control group^34,35^. In the current study, this approach was used to examine available RCTs to again explore the efficacy of pharmacological therapies including tamsulosin, doxazosin, nifedipine, terazosin and rowatinex in promoting the expulsion of urinary stone fragments after ESWL. More importantly, we subdivided the available data according to follow-up duration, and then compared whether there were differences between those medications throughout the period of stone expulsion.

## Materials and Methods

The present systematic review was performed in accordance with the latest Preferred Reporting Items for Systematic Reviews and Meta-Analysis Statement^36^.

### Search strategy and selection criteria

**Figure 1:**
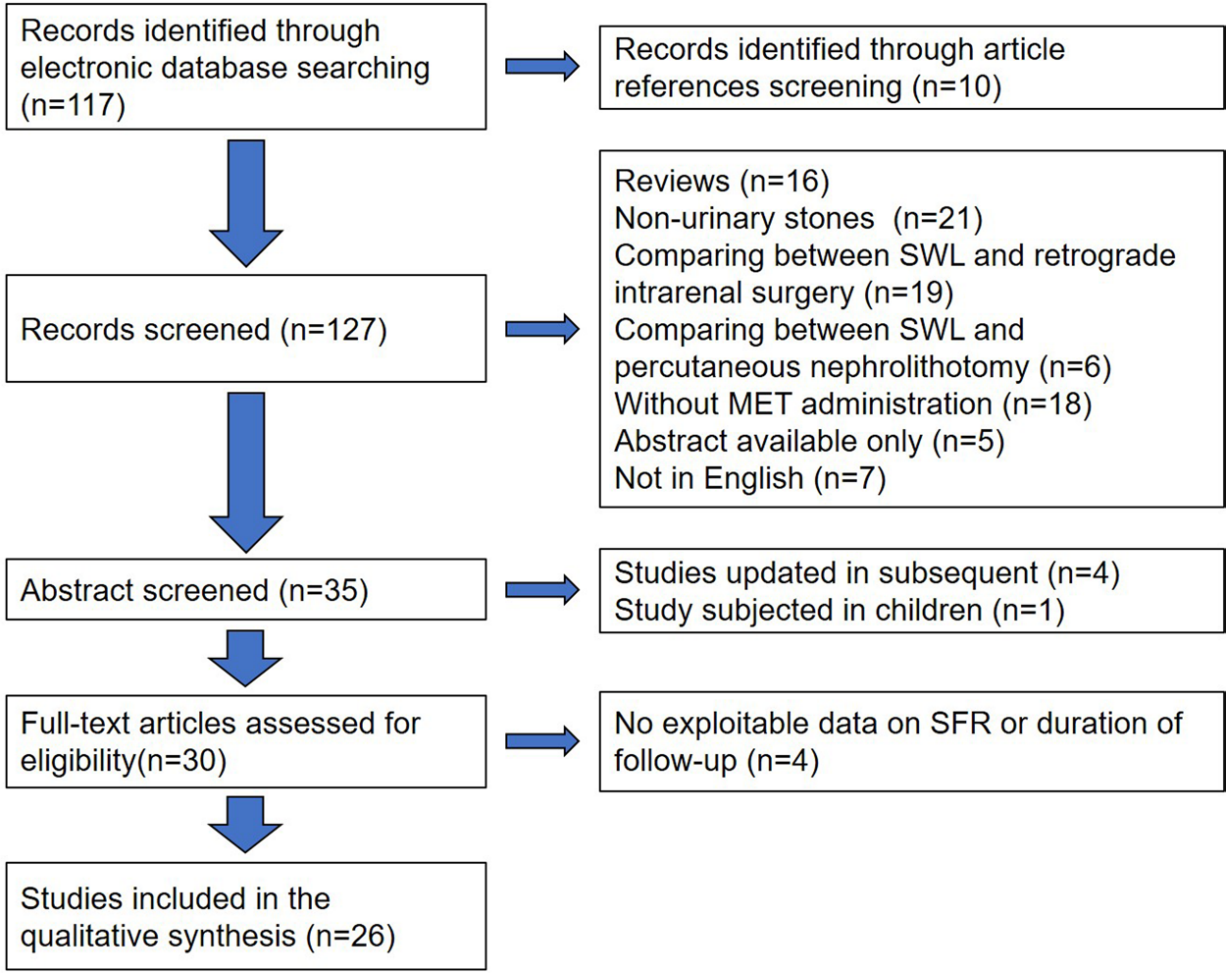
PRISMA study flow diagram.

We searched the US National Library of Medicine’s life science database (Medline), Embase, the Cochrane Central Register of Controlled Trials and the Cochrane Database for Systematic Reviews for articles published before March 2016. The language of publication was limited to English. Any of the terms ‘calcium-channel blocker’, ‘adrenergic alpha-antagonist’, ‘prostaglandin antagonist’, ‘prostaglandin’, ‘cortisone’, ‘nifedipine’, ‘verapamil’, ‘diltiazem’, ‘tamsulosin’, ‘terazosin’, ‘doxazosin’, ‘alfuzosin’, ‘prazosin’, ‘deflazacort’, ‘prednisone’, ‘medical therapy’, ‘drug therapy combination’, ‘medical management’, ‘expulsive therapy’, ‘facilitated passage’ and ‘adjuvant medical expulsive therapy’ was used in conjunction with any of the terms ‘urolithiasis’, ‘lithotripsy’, ‘extracorporeal shock wave lithotripsy’, ‘renal’, ‘urinary’ and ‘ureteral’. And the terms of ‘randomized controlled trial’ or ‘random’ was imposed as a restriction to the searching process. The search flowchart is presented in Fig. 1 ^8–33^.

Studies that met all of the following criteria were included: (1) randomized controlled trials on the medical therapy for patients undergoing ESWL for renal or ureteral calculus; (2) studies reported the SFR and duration of follow-up; (3) after stone expulsion or at the end of the follow-up period, radiologic evaluation was mandatory to confirm stone passage; and (4) loss of follow-up rate < 10%. Moreover, (5) crossover trials, dose titration studies, daily dosing studies and studies that were only available as abstracts were excluded.

### Outcome measures and data extraction

The data were extracted by two independent reviewers (Yang and Chen). A third reviewer resolved any disagreements (Liao). We extracted trial design; trial size; details of intervention including dose and treatment duration; and patient characteristics such as mean age, sex, mean duration of symptoms, duration of follow-up, type of outcome (number of patients with successful stone expulsion) and outcome data at each duration of follow-up.

### Risk of bias assessment

Two review authors (Yang and Chen) independently evaluated all relevant clinical studies for methodological quality. Each review author performed this assessment using The Cochrane Collaboration’s Risk of Bias tool, which included quality of random allocation concealment, description of dropout and withdrawal, intention-to-treat analysis, and blinding procedures for treatment and outcome assessments^37^. A third reviewer resolved any disagreements by discussion (Liao). We synthesized qualitative information using Review Manager, version 5.3 (Copenhagen: The Nordic Cochrane Centre, The Cochrane Collaboration, http://tech.cochrane.org/revman).

### Statistical analysis

The primary outcome was the number of patients with successful stone expulsion before the time point of interest. We subdivided the outcome data into three groups according to the duration of follow-up as follows: ≤15 days (group 1); >15 but ≤45 days (group 2); and >45 but ≤90 days (group 3).

Statistical analysis was performed with Stata SE 14 (Stata Corp, College Station, TX, USA). A standard network model for two-category data was established and consistency analysis was performed in each subgroup. Primary outcomes were gauged by the standard network model; the mean differences with 95% CIs (confidence interval) of each intervention compared with control or any two interventions compared with each other were worked out. If the 95% CI was above or under than 1.00, then the difference was statistically significant (*p* < 0.05).

We used the surface under the cumulative ranking (SUCRA) probabilities to assess the efficacy of different drugs, which is a common method used in network meta-analysis. SUCRA expresses a percentage representing the efficacy of every intervention compared with an imaginary intervention that is the best without uncertainty. A higher SUCRA score indicates a more probability to be effective.

Tau was used to assess the risk of bias in each model. Tau < 1 means that there is a low risk of bias in the model.

## Result

**Figure 2:**
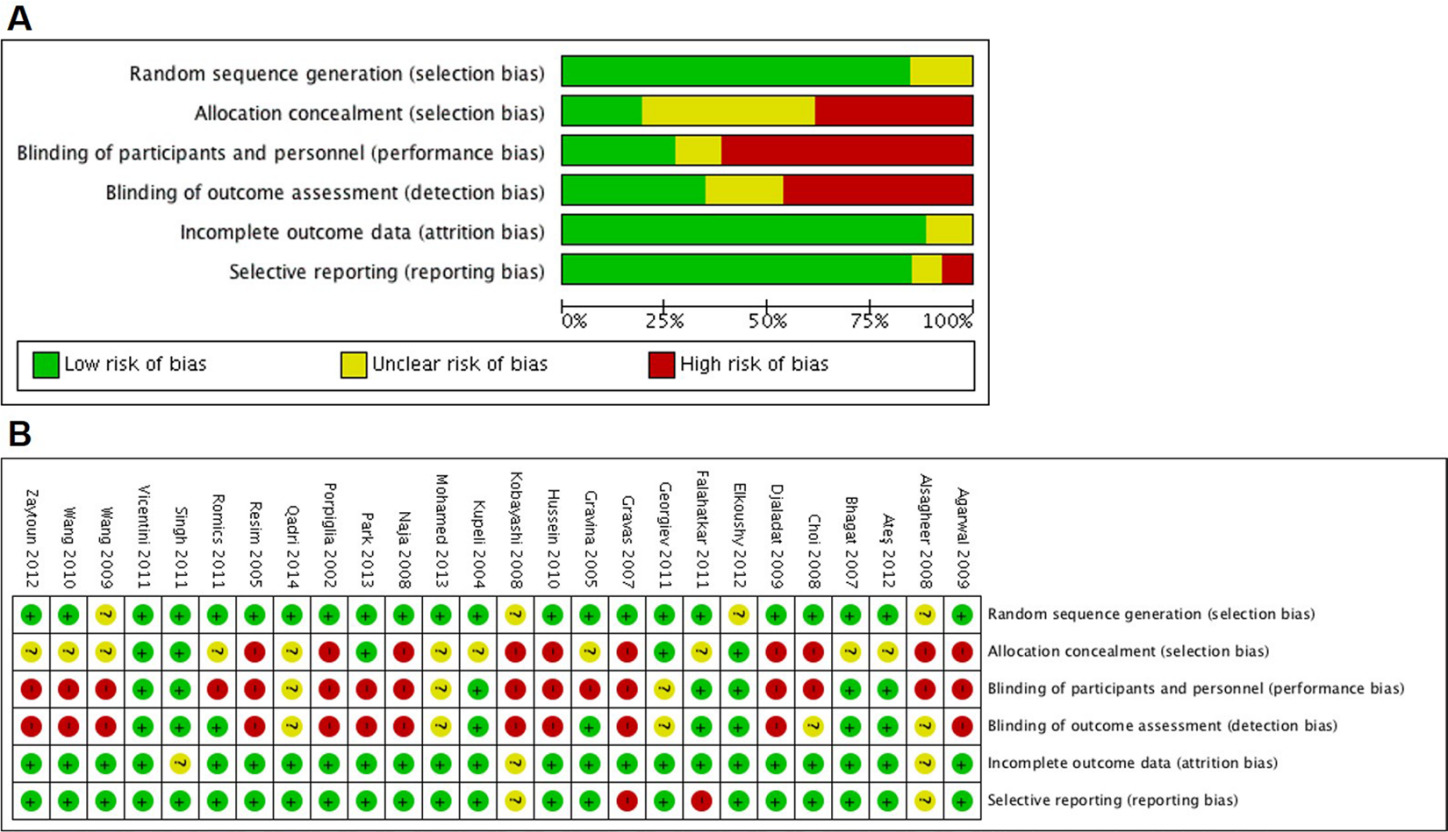
Risk-of-bias analysis: (**A**) Risk of bias graph: review authors’ judgements about each risk of bias item presented as percentages across all included studies. (**B**) Risk of bias summary: review authors’ judgements about each risk of bias item for each included study.

The final analysis here included 26 studies with 5 interventions (tamsulosin, terazosin, doxazosin, nifedipine and rowatinex) compared with placebo or non-placebo on a total of 2775 patients^8–33^. Ten studies reported expulsion results with follow-up duration ≤15 days (Table 1), for 16 studies the follow-up duration was >15 but ≤45 days (Table 2), and for 10 studies it was >45 but ≤90 days (Table 3). The details of the risk of bias are shown in Fig. 2.

**Table 1:** Characteristics of included studies which the durations of follow-up were less than or equal to 15 days.

Author, year	Pts(n)	research region	Gender (male, n)	Age (years)	Stone location	Stone size, mm	SWL sessions	Treatment	SFRs, %	Follow-up
Ateş, 2012	35/44	Turkey	25/33	38.4/31.0	Upper ureteral	9.06/8.30	1.26/1.23	Doxazosin 4 mg/ non-Placebo	94/80	2 weeks
Romics, 2011	106/98	Germany	62/53	51/48	Renal	3–20	1/1	Rowatinex 300 mg/Placebo	21/14	1 weeks
Hussein, 2010	67/69	Egypt	40/45	44/40	Renal	4–24	1–2	Tamsulosin 0.4 mg/non-Placebo	15/12	2 weeks
Wang, 2010	55/52	China	NA	42.2/40.9	Lower ureteral	9.3/8.6	1/1	Tamsulosin 0.4 mg/Placebo	75/46	2 weeks
Djaladat, 2009	50/50	Iran	30/29	38.3/40.9	Renal	10–20	1/1	Rowatinex 300 mg/Placebo	18/4	2 weeks
Wang, 2009	34/35/38	China	22/22/25	50.9/51.5/51.9	Lower ureteral	6.6/6.4/6.7	1/1/1	Tamsulosin 0.4 mg/Terazosin 2 mg/Placebo	85/80/82	2 weeks
Agarwal, 2009	20/20	India	15/16	32.4/35.5	Upper ureteral	9.4/10.4	1.6/2.0	Tamsulosin 0.4 mg/non-Placebo	55/25	15 days
Choi, 2008	32/31/33	Korea	25/26/25	48.0/45.2/45.9	Ureteral	7.6/7.3/7.4	1/1/1	Tamsulosin 0.2 mg/ Nifedipine 30 mg/non-Placebo	84/68/61	2 weeks
Gravas, 2007	30/31	Greece	18/20	48.8/49.2	Distal ureteral	8.3/8.5	1/1	Tamsulosin 0.4 mg/non-Placebo	53/45	2 weeks
Kupeli, 2004	24/24	Turkey	NA	42.7/43.1	Lower ureteral	8.2/8.6	1/1	Tamsulosin 0.4 mg/non-Placebo	71/33	15 days

Stone size and SWL sessions was presented as the mean or range value.

Pts = patients; SWL = shock wave lithotripsy; SFRs = stone-free rates; NA = not available.

**Table 2:** Characteristics of included studies which the durations of follow-up were greater than 15 days but less than or equal to 45 days.

Author, year	Pts(n)	research region	Gender (male, n)	Age(years)	Stone location	Stone size, mm	SWL sessions	Treatment	SFRs, %	Follow-up
Mohamed, 2013	65/65	Egypt	41/39	40.1/43.8	Ureteral	5–15	1–3	Tamsulosin 0.4 mg/non-Placebo	85/89	30 days
Park, 2013	44/44	Korea	29/28	46.2/47.6	Proximal ureteral	9.2/9.6	1/1	Tamsulosin 0.2 mg/non-Placebo	84/66	3 weeks
Elkoushy, 2012	63/63	Egypt	44/39	52.8/49.4	Renal and upper ureteral	11.1/10.5	1.5/1.9	Tamsulosin 0.4 mg/non-Placebo	55/46	3 weeks
Georgiev, 2011	99/87	Bulgaria	67/54	54/51	Renal and ureteral	5–20	1–2	Tamsulosin 0.4 mg/non-Placebo	74/56	4 weeks
Romics, 2011	106/98	Germany	62/53	51/48	Renal	3–20	1/1	Rowatinex 300 mg/Placebo	44/30	4 weeks
Singh, 2011	59/58	India	44/41	32.2/36.0	Upper ureteral	6–15	1/2	Tamsulosin 0.4 mg/non-Placebo	85/71	1 months
Vicentini, 2011	38/35/38	Brazil	16/18/24	47.3/48.6/45.7	Renal	10/10/12	1/1/1	Tamsulosin 0.4 mg/ Nifedipine 20 mg/Placebo	61/49/37	30 days
Hussein, 2010	67/69	Egypt	40/45	44/40	Renal	4–24	1–2	Tamsulosin 0.4 mg/non-Placebo	46/32	1 months
Djaladat, 2009	50/50	Iran	30/29	38.3/40.9	Renal	10–20	1/1	Rowatinex 300 mg/Placebo	24/18	4 weeks
Kobayashi, 2008	38/34	Japan	NA	56.8/52.3	Ureteral	10.61/9.85	1/1	Tamsulosin 0.2 mg/non-Placebo	84/88	28 days
Naja, 2008	51/65	India	36/43	37.1/39.4	Renal	12.1/13.1	1.66/2.16	Tamsulosin 0.4 mg/non-Placebo	53/31	3 weeks
Bhagat, 2007	29/29	America	22/24	35.9/42.3	Renal and ureteral	6–24	1/1	Tamsulosin 0.4 mg/Placebo	97/79	30 days
Gravas, 2007	30/31	Greece	18/20	48.8/49.2	Distal ureteral	8.3/8.5	1/1	Tamsulosin 0.4 mg/non-Placebo	63/52	4 weeks
Resim, 2005	32/35	Turkey	21/22	39/37	Lower ureteral	21/20	N/A	Tamsulosin 0.4 mg/non-Placebo	75/66	6 weeks
Gravina, 2005	65/65	Italy	28/29	48.4/47.9	Renal	14.2/14.6	1/1	Tamsulosin 0.4 mg/non-Placebo	55/46	4 weeks
Porpiglia, 2002	40/40	Italy	27/25	50/46	Ureteral	11.6/10.1	1/1	Nifedipine 30 mg/non-Placebo	75/50	45 days

Stone size and SWL sessions was presented as the mean or range value.

Pts = patients; SWL = shock wave lithotripsy; SFRs = stone-free rates; NA = not available.

**Table 3:** Characteristics of included studies which the durations of follow-up were greater than 45 days but less than or equal to 90 days.

Author, year	Pts(n)	research region	Gender (male, n)	Age(years)	Stone location	Stone size, mm	SWL sessions	Treatment	SFRs, %	Follow-up
Qadri, 2014	60/60	Pakistan	41/48	39/41	Renal	11.2/10.5	1/1	Tamsulosin 0.4 mg/non-Placebo	97/80	8 weeks
Zaytoun, 2012	50/50/50	France	25/31/23	39.4/39.2/40.5	Renal	16.6/16.1/15.9	2.02/2.12/2.08	Tamsulosin 0.4 mg/ Doxazosin 4 mg/non-Placebo	92/90/84	3 months
Georgiev, 2011	99/87	Bulgaria	67/54	54/51	Renal and ureteral	5–20	1–2	Tamsulosin 0.4 mg/non-Placebo	91/75	12 weeks
Romics, 2011	106/98	Germany	62/53	51/48	Renal	3–20	1/1	Rowatinex 300 mg/Placebo	68/50	12 weeks
Singh, 2011	59/58	India	44/41	32.2/36.0	Upper ureteral	6–15	1/2	Tamsulosin 0.4 mg/non-Placebo	92/86	3 months
Falahatkar, 2011	70/71	Iran	53/52	45.5/47.0	Renal and ureteral	13.22/12.88	1/1	Tamsulosin 0.4 mg/Placebo	71/61	12 weeks
Hussein, 2010	67/69	Egypt	40/45	44/40	Renal	4–24	1–2	Tamsulosin 0.4 mg/non-Placebo	73/55	3 months
Alsagheer, 2008	52/53	Egypt	NA	35.2/33.6	Renal and upper ureteral	5–20	1–4	Doxazosin 1 mg/non-Placebo	92/75	12 weeks
Naja, 2008	51/65	India	36/43	37.1/39.4	Renal	12.1/13.1	1.66/2.16	Tamsulosin 0.4 mg/non-Placebo	94/85	12 weeks
Gravina, 2005	65/65	Italy	28/29	48.4/47.9	Renal	14.2/14.6	1/1	Tamsulosin 0.4 mg/non-Placebo	78/60	12 weeks

Stone size and SWL sessions was presented as the mean or range value.

Pts = patients; SWL = shock wave lithotripsy; SFRs = stone-free rates; NA = not available.

The average SFRs for the included studies in group 1, 2 and 3 were 43.5%, 59.2% and 75.9%, respectively. The intervention studied by most trials was tamsulosin with 21 studies of 1025 patients, while doxazosin, nifedipine, terazosin and rowatinex were examined in 3 studies of 137 patients, 3 studies of 106 patients, 1 study of 35 patients and 2 studies of 156 patients, respectively. Placebo was employed in 7 studies of 439 patients, while the other 19 studies of 877 patients had a non-placebo control.

The average SFRs for placebo in group 1, 2 and 3 were 29.8%, 43.5% and 54.4%, while those for non-placebo were 40.7%, 57.0% and 72.6%, respectively. As for each medical therapy, the average SFRs for tamsulosin were 57.6%, 72.1% and 84.8%, while those for rowatinex were 19.9%, 37.8% and 67.9% in the same groups, respectively. The results for terazosin in group 2 and 3, doxazosin in group 2, and nifedipine in group 3 were not available, so only the available results were analysed. Terazosin had an average SFR of 80% in group 1, doxazosin had ones of 94.3% and 91.2% in group 1 and 3, and nifedipine had ones of 67.7% and 62.7% in group 1 and 2, respectively.

A preliminary network meta-analysis was conducted among tamsulosin, placebo and non-placebo interventions without subdivision according to the time point of interest, to determine whether the placebo and non-placebo interventions could be pooled together. The results showed that there was no significant difference between placebo and non-placebo (*p* > 0.05). The SUCRA ranking was: tamsulosin > placebo > non-placebo (99.4, 0.6 and 0, respectively). Then, the following analyses were conducted using a control group that consisted of the placebo and non-placebo interventions.

**Figure 3:**
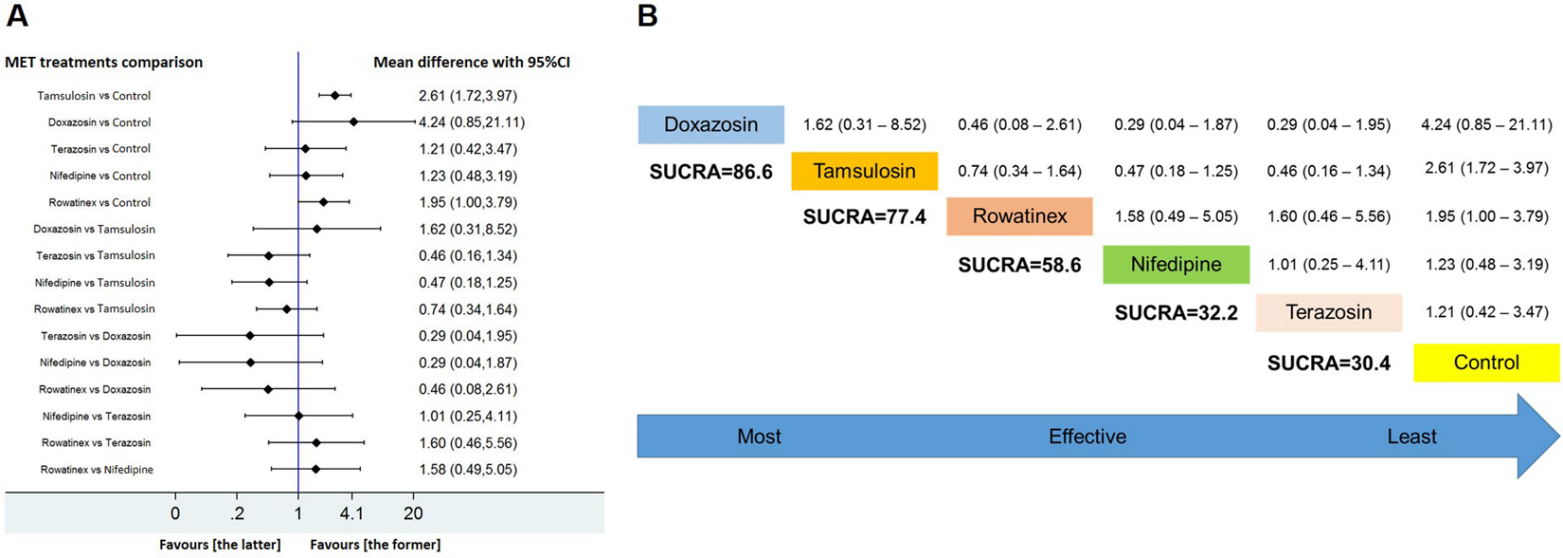
Network meta-analysis and SUCRA rank of included medications for improving SFR after ESWL which the endpoint of revisit was less than or equal to 15 days. Tamsulosin, doxazosin, terazosin, nifedipine, rowatinex and control represented the outcome that was extracted and pooled from included studies within this subdivided period respectively. The forest plot (**A**) showed each MET treatment compared with control group or any two treatments compared with each other, and the mean difference with 95% CI of each comparison. Those mean differences greater than 1 favored the former one of the comparison, and those mean differences less than 1 favored the latter one. If the 95% CI was above or under than 1.00, the difference was statistically significant (*p* < 0.05). The SUCRA ranks were calculated through the standard network model and listed in order from high to low (**B**).

The network meta-analyses were conducted in each subgroup. In group 1, doxazosin had the highest SUCRA rank which was the first possibility to be effective; however, its mean difference was not statistically significant when compared with control (*p* > 0.05), since a relative small number of patients were included in doxazosin intervention resulting in a relative large scale of 95% CI. Tamsulosin and rowatinex had the second and third SUCRA rank and their mean difference were statistically significant when compared with control (*p* < 0.05). Terazosin and nifedipine had lowest SUCRA rank which were the least likely to be effective. The SUCRA outcome showed the following efficacy ranking: doxazosin > tamsulosin > rowatinex > nifedipine > terazosin > control (Fig. 3).

**Figure 4:**
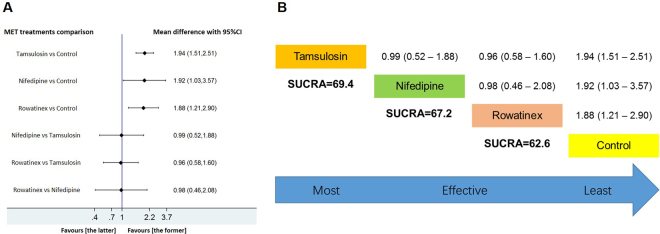
Network meta-analysis and SUCRA rank of included medications for improving SFR after ESWL which the endpoint of revisit was greater than 15 days but less than or equal to 45 days. Tamsulosin, nifedipine, rowatinex and control were involved in the forest plot (**A**) and the SUCRA rank calculation (**B**). If the 95% CI was above or under than 1.00, the difference was statistically significant (*p* < 0.05).

In group 2, all involved interventions (tamsulosin, nifedipine and rowatinex) had SFRs that were significantly better than that in the control group (*p* < 0.05). They had almost the same SUCRA rank with similar possibility to be effective (Fig. 4).

**Figure 5:**
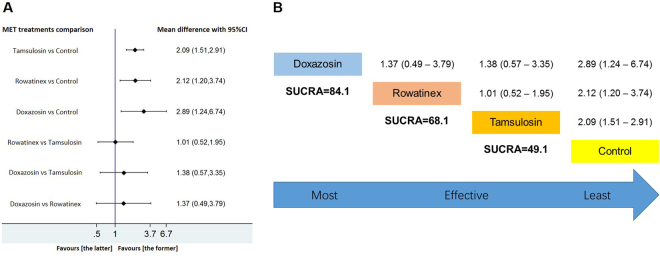
Network meta-analysis and SUCRA rank of included medications for improving SFR after ESWL which the endpoint of revisit was greater than 45 days but less than or equal to 90 days. Tamsulosin, doxazosin, rowatinex and control were involved in the forest plot (**A**) and the SUCRA rank calculation (**B**). If the 95% CI was above or under than 1.00, the difference was statistically significant (*p* < 0.05).

In group 3, all involved interventions (tamsulosin, doxazosin and rowatinex) were associated with significantly better outcomes compared with control (*p* < 0.05). Doxazosin had the highest SUCRA rank which was the first possibility to be effective; rowatinex and tamsulosin had the second and third SUCRA rank indicating that they were less likely to be effective compared with doxazosin. The SUCRA ranking was: doxazosin > rowatinex > tamsulosin > control (Fig. 5).

The risk bias was low in all of the above network meta-analyses (tau² < 0.001). No consistency problems were detected in this model (Appendix 3).

## Discussion

Overcoming the absence of head-to-head comparisons between pharmacological therapies in promoting the expulsion of urinary stone fragments after ESWL in existing studies, our network meta-analysis of 26 RCTs provides evidence for the efficacy of commonly used medications for improving SFR after ESWL. Among these, doxazosin showed a superior recommendation level (SUCRA rank) over all other medications at the follow-up durations of ≤15 days and 45–90 days. Doxazosin has the potential to be considered as the first choice of pharmacological therapy for improving SFR after ESWL. Unfortunately, the effect of doxazosin at time point of 15–45 days was not assessed because of a lack of available data for statistical analysis. Tamsulosin and rowatinex were shown to be effective for improving SFR after ESWL at all three time point, but the results did not support tamsulosin or rowatinex as the best choice due to relatively low recommendation levels. In addition, tamsulosin seemed to have a decreased trend of efficacy over the duration of follow-up; its SUCRA score dropped from 77.4 at 15 days to 49.1 at 90 days, which represented a change from being higher than that of rowatinex to being lower than it. Nifedipine did not show effectiveness at a time point of less than 15 days, but could improve the SFR after ESWL at 45 days, almost the same as tamsulosin, indicating that the relaxation and dilation effects of nifedipine on ureteral smooth muscle were slow. Terazosin did not show effectiveness at a follow-up duration of less than 15 days with only one included RCT, which studied lower ureteral stone MET after ESWL. As a pharmacological therapy for upper ureteral or renal stone fragments after ESWL, the efficacy of terazosin is unknown.

These results provide a very wide perspective about the expulsion of stone fragments after ESWL with or without medications. Normally, stone fragments generated by ESWL are smaller than 4 mm and tend to pass spontaneously through the ureter without the influence of any drug^14^. The fragment size is an important factor that determines the passage of a stone through the narrowest part of the ureter^38–40^. If the stone fragments are slightly larger, then the expulsion is often accompanied by colic and lower urinary tract symptoms, sometimes lasting several days, which cause discomfort. If the stone fragments are larger than 6 mm, or any ureteral status such as oedema or spasm occurs, the fragments may not pass the ureter and repeat treatment or a secondary procedure will be needed. For the two hypothetical conditions above, MET after ESWL provides two possible sources of efficacy: accelerating the process of expulsion or improving the SFR.

A single RCT often focuses on the effectiveness of one or two medications within a certain period. According to its design, it can precisely resolve the issue of whether these medications can improve the SFR compared with a placebo or watchful waiting. However, what if a medication can only accelerate the process of expulsion in a short period such as 15 days, but does not improve the SFR in a long period such as 30 days or more? In this case, an analysis for a short time will lead to the conclusion that the medication worked, but an analysis at the end will result in the conclusion that it was useless. Pickard and colleagues^7^ used the double-blind, randomized, controlled method to the extreme, taking outcomes that were closest to clinical practice, leading to the conclusion that the drugs were ineffective for stone expulsion at the time of the outcome, but neglected the possibility that the drugs may result in acceleration of the process of expulsion before the endpoint of 4 weeks. Here, it should be pointed out that the effect of accelerating the process of expulsion should also be considered to represent efficacy because it can reduce the duration of discomfort and symptoms, such as lower urinary tract symptoms and pain, and may even reduce the incidence of complications such as steinstrasse. In our study, available data of the use of medications for improving SFR after ESWL were pooled together. We attempted to subdivide these results into different follow-up durations, taking advantage of network meta-analysis to indirectly compare multiple treatments, which can better explain the existing controversy about MET and provide a guide for using medications after ESWL.

Selective α1-adrenoceptor antagonist has a certain rapid effect for promoting the expulsion of stone fragments. It is well established that the density of α1-adrenoceptors is higher in the distal ureter^3,41^. Studies have also shown that tamsulosin acts on α_1A_ and α_1D_ receptors in the lower ureter, prevents spasm by relaxing the smooth muscle of the ureter, reduces proximal ureteral pressure and acts on the C-fibres blocking pain conduction^42,43^. Moreover, Tamsulosin has little effect on blood pressure, while doxazosin and terazosin can lead to orthostatic hypotension^44^; this is because tamsulosin is highly selective for the α_1A_ receptor, which is specifically located in the urinary system, while doxazosin and terazosin have low selectivity for α1-adrenoceptor subtypes. The current study demonstrated that doxazosin had a higher SUCRA score than tamsulosin for improving SFR after ESWL. A possible hypothesis to explain this is that high selectivity on α1-adrenoceptor subtypes of tamsulosin may result in lower efficacy of blocking than subtypes of doxazosin with low selectivity. Our data indicate that doxazosin can improve the SFR after ESWL, while tamsulosin may result more in acceleration of the process of expulsion after ESWL. This is because the SUCRA rank of tamsulosin dropped from 77.4 at 15 days to 49.1 at 90 days, indicating that more stone fragments may be passed spontaneously in the long term without the influence of medication. Our data also demonstrated that terazosin had no impact on improving SFR after ESWL at 15 days; however, more well-designed RCTs should be performed to investigate the efficacy and safety of doxazosin and terazosin for promoting the expulsion of stone fragments.

Calcium channel blockers have also received wide attention for their efficacy at promoting the expulsion of urinary stones, because smooth muscle contraction is directly caused by an increase in calcium concentration. Nifedipine also relaxes the ureter^45^, leading to less pain^14^, but Davenport and colleagues^46^ performed an interesting study showing that nifedipine could not reduce the contraction frequency of the ureter and could not maintain ureteric pressure at a relatively low level as well as tamsulosin could. The current consensus is that nifedipine has an effect of promoting the expulsion of ureteral stones but is not superior to tamsulosin, either in improving SFR or in reducing expulsion time^47^. Our data confirmed that nifedipine could not improve the SFR after ESWL at a follow-up duration of less than 15 days, but was effective upon drug therapy for more than 30 days. This indicated that the relaxing and dilatory effects of nifedipine on ureteral smooth muscle occurred later than for α-adrenoceptor antagonist, meaning that nifedipine may have less potential to reduce the discomfort of patients who are suffering ureteral calculus compared with tamsulosin.

Rowatinex is an essential oil preparation of terpene composed of pinene (3%), camphene (15%), borneol (10%), anethol (4%) and cineol (3%) in olive oil, which has been suggested for the treatment of urolithiasis and other urological problems^22,48^. It was assumed to improve renal blood flow, thus giving rise to increased urine excretion, and to have antispasmodic effects to facilitate the passage of urinary stones^22^. In contrast to other medications, the mechanism of rowatinex for promoting the expulsion of stone fragments is a combined effect and cannot be clearly outlined; nevertheless, clinical data showed that it is not only a valuable drug used for urolithiasis, but also has spasmolytic and anti-inflammatory properties^16^. The current study demonstrated that rowatinex had a relatively stable SUCRA score from 1-week to 12-week follow-up after ESWL. It was even better than tamsulosin at the time point of 90 days after ESWL, but more studies are needed to investigate that effectiveness further.

Some limitations of the present network meta-analysis should be mentioned: (1) The outcome data were subdivided into three groups according to the follow-up duration with the intent of exploring the effect of these medications over time. However, there were no data for terazosin at 45 and 90 days, doxazosin at 45 days and nifedipine at 90 days after ESWL in the existing literature. Hence, the conclusion that doxazosin has potential to be the first choice or to be better than other medications for improving SFR after ESWL still requires more well-designed RCTs to be confirmed. In addition, the long-term efficacy of nifedipine or terazosin as MET after ESWL is still unclear. (2) Among 2775 patients in 26 RCTs that we included, 1025 patients in 21 RCTs were assigned to tamsulosin intervention, while only limited studies focused on doxazosin, nifedipine, terazosin and rowatinex, with only 137 patients in 3 RCTs, 106 patients in 3 RCTs, 35 patients in 1 RCT and 156 patients in 2 RCTs, respectively. Thus, because of the weight relationship in network meta-analysis, the outcome of interventions with very few objects may be affected by calculating with an intervention with vast objects through the network meta-analysis model. Therefore, more studies are needed before a definitive conclusion about the effect of MET after ESWL can be drawn. (3) Although no problems with consistency were detected here, the SFR ranged from 4% to 89% in the control group and from 15% to 97% under medical interventions. The reason for this was that cases with a wide range of locations of stones from the kidney to the distal ureter were included, and different criteria regarding how stone clearance was defined were used in different studies. Thus, a potential for bias may have been generated.

## Conclusion

The current network meta-analysis demonstrated that tamsulosin, doxazosin, nifedipine and rowatinex are effective for promoting the expulsion of urinary stone fragments after ESWL, of which α-adrenoceptor antagonists are better than calcium channel blockers, especially at a short expulsion time. Among three types of α-adrenoceptor antagonists, doxazosin can improve the SFR after ESWL in the long term, while tamsulosin may result more in accelerating the process of expulsion after ESWL, but terazosin did not show any efficacy, at least in the existing literature. Therefore, doxazosin and tamsulosin have the potential to be considered as the first choices of pharmacological therapy to improve SFR after ESWL, although more high-quality RCTs will be needed to evaluate the efficacy and reliability of doxazosin compared with tamsulosin.

## Electronic supplementary material

supplementary information
